# Regional variation of potentially avoidable hospitalisations in Switzerland: an observational study

**DOI:** 10.1186/s12913-021-06876-5

**Published:** 2021-08-21

**Authors:** Niklaus Gygli, Franziska Zúñiga, Michael Simon

**Affiliations:** 1 Faculty of Medicine, Department of Public Health, Institute of Nursing Science, University of Basel, Bernoullistr. 28, CH-4056 Basel, Switzerland; 2 Department of Nursing, University Hospital Basel, Spitalstrasse 21, CH-4031 Basel, Switzerland; 3 Nursing and Midwifery Research Unit, Department of Nursing, University Hospital Bern, Freiburgstrasse 18, CH-3010 Bern, Switzerland

**Keywords:** Potentially avoidable hospitalisations, Ambulatory care sensitive conditions, Switzerland, Primary health care, Regional variation

## Abstract

**Background:**

Primary health care is subject to regional variation, which may be due to unequal and inefficient distribution of services. One key measure of such variation are potentially avoidable hospitalisations, i.e., hospitalisations for conditions that could have been dealt with in situ by sufficient primary health care provision. Particularly, potentially avoidable hospitalisations for ambulatory care-sensitive conditions (ACSCs) are a substantial and growing burden for health care systems that require targeting in health care policy.

**Aims:**

Using data from the Swiss Federal Statistical Office (SFSO) from 2017, we applied small area analysis to visualize regional variation to comprehensively map potentially avoidable hospitalisations for five ACSCs from Swiss nursing homes, home care organisations and the general population.

**Methods:**

This retrospective observational study used data on all Swiss hospitalisations in 2017 to assess regional variations of potentially avoidable hospitalisations for angina pectoris, congestive heart failure, chronic obstructive pulmonary disease, diabetes complications and hypertension. We used small areas, utilisation-based hospital service areas (HSAs), and administrative districts (Cantons) as geographic zones. The outcomes of interest were age and sex standardised rates of potentially avoidable hospitalisations for ACSCs in adults (> 15 years). Our inferential analyses used linear mixed models with Gaussian distribution.

**Results:**

We identified 46,479 hospitalisations for ACSC, or 4.3% of all hospitalisations. Most of these occurred in the elderly population for congestive heart failure and COPD. The median rate of potentially avoidable hospitalisation for ACSC was 527 (IQR 432–620) per 100.000 inhabitants. We found substantial regional variation for HSAs and administrative districts as well as disease-specific regional patterns.

**Conclusions:**

Differences in continuity of care might be key drivers for regional variation of potentially avoidable hospitalisations for ACSCs. These results provide a new perspective on the functioning of primary care structures in Switzerland and call for novel approaches in effective primary care delivery.

**Supplementary Information:**

The online version contains supplementary material available at 10.1186/s12913-021-06876-5.

## Background

Primary health care is vital to population health. However, regional variability of its quality and accessibility may lead to unequal and inefficient distribution of services. One key measure of this variability is the rate of avoidable hospitalizations, i.e., those for conditions that could have been treated with appropriate chronic disease management in place in primary health care [1–3]. In addition to incurring considerable extra costs, treating these conditions in hospital disrupts the affected patients’ normal care provision [4, 5].

Ambulatory care-sensitive conditions (ACSC) support the measurement and comparison of rates of potentially avoidable hospitalisations. These were originally defined by international organisations such as the Organisation for Economic Co-operation and Development (OECD) and further developed by scientific experts [3]. Potentially avoidable hospitalisations for ACSCs are most common in the elderly population and increase with age [6, 7]. Chronic conditions account for up to 60% of total potentially avoidable hospitalisations and their respective costs [6, 7]. Additionally, ACSCs account for up to 48% of annual emergency department visits and 20% of overall hospitalisations [7–10]. The total cost of potentially avoidable hospitalisations for ACSCs are a growing economic burden for health care systems [6, 7, 11]. Approximately 42% of hospitalized nursing home residents are referred for ACSCs, thus generating unnecessary costs of up to 102 million Swiss francs annually in Switzerland [12]. Furthermore, there is a socio-economic gradient, with lower ACSC rates in higher income regions [13] However, it is unclear how different settings and respective primary healthcare providers handle ACSC. Therefore, especially for the elderly population, it is vital not only to assess total rates of avoidable ACSC-based hospitalisations, but also to differentiate between the involved settings.

We identified five conditions that are both commonly used in health services research literature and highly prevalent amongst chronically ill populations [1–3, 5–7, 10]. In descending order of occurrence, these are: congestive heart failure, chronic obstructive pulmonary disease (COPD), diabetes complications, hypertension and angina pectoris.

A large proportion of hospitalisations for these conditions are deemed preventable, i.e., they can normally be positively influenced via effective chronic disease management by primary care providers [10].

Switzerland offers a unique opportunity to explore regional variations in potentially avoidable hospitalisations. Because of its status as a confederation, Swiss health law includes relatively high levels of regional autonomy, allowing regions to establish their own care structures and approaches [14]. Mandatory health insurance with varying deductibles and primary care—mostly provided by general practitioners with freedom of choice for patients—provides the basis for all administrative districts [14]. Activity-based funding to reimburse hospitals has been used since 2012 [15].

Overall, the Swiss primary health care system is stable and fully functional and its income inequality quite low [2, 16–19]. Switzerland is the world’s second-highest per capita spender on health care [2, 16–19], and considering that income is relatively equally distributed across the country, administrative districts are offered similar preconditions to establish their primary care structures. A recent study of ACSC Swiss contexts showed a 12-fold level of variation among small regions. That particular study was restricted to certain regions of Switzerland and provided limited insight into regional patterns regarding diagnoses and primary care provision in different settings [2].

Employing small area analysis with data from the Swiss federal statistical office, we sought to establish the first complete epidemiological map of potentially avoidable hospitalisations for our five selected ACSCs from Swiss nursing homes, home care organisations and the general population. Detailed information on the operationalization of terms such as hospitalization or small area used by the Swiss federal statistical office are provided in supplementary file A. A list explaining all abbreviations used is provided in supplementary file B.

## Method

### Design and sample

This retrospective analysis used routine health care data of all Swiss hospitals [20] from 2017, as it was the most recent dataset provided by the Swiss Federal Statistical Office (SFSO) containing all hospitalizations in Switzerland. To assess the suitability of eligible diagnoses we used a set of quality indicators provided by the OECD to compare the quality of health care provision between countries. As the OECD Health Care Quality Indicator Project uses potentially avoidable hospitalisations for ACSCs as a quality measure for chronic disease management in primary care [3, 21], we included four prominent chronic conditions from their list of indicators: congestive heart failure, chronic obstructive pulmonary disease (COPD), diabetes complications and hypertension [3, 21]. A fifth indicator, angina pectoris, is commonly used in similar settings [6, 7]. ICD 10 codes for these conditions which were used can be found in Table 1. Consistent with the OECD criteria, we included all hospitalisations in the population aged 15+ and not referred from other hospitals or rehabilitation clinics. All hospitalisations with a main diagnosis of the above mentioned ICD-10 Codes were considered avoidable [3, 21]. Data for this study was provided by the SFSO based on a data protection contract in accordance with article 22 of the Swiss Federal Act on data protection. The sample consists of routinely collected and de-identified data and therefore exempt from ethics approval.

**Table 1:** ICD 10 Codes and Sources for Analysis

*ICD 10 Codes and Sources for Analysis*		
Condition	ICD-10 Codes	Source
Angina pectoris	I20 I24.0 I24.8 I24.9	Purdy et al.
Congestive heart failure	I11.0 I50 J81	OECD
COPD	J20 J40 J41 J42 J43 J44 J47	OECD
Diabetes Complications	E10.0-E10.8 E11.0-E11.8 E12.0-E12.8 E13.0-E13.8 E14.0-E14.8	OECD
Hypertension	I10 I11.9	OECD

### Data sources

Data for this study were extracted from the SFSO’s annual census report and from the medical statistical data collected by Swiss hospitals on all hospitalisations. Census data and the medical statistical data was linked on the small area level. Census data included age and gender distribution for each small area [22]. All Swiss hospitals collect medical data continuously in compliance with Swiss federal law and provide them annually to the SFSO [23]. Patients hospitalised multiple times were assigned to multiple cases with unique anonymized patient identifiers to allow us to track their hospital admissions throughout the year. Due to the exhaustive nature of the dataset, we did not expect a biased dataset.

### Geographic areas

We used the Swiss acute care hospitals’ and administrative districts’ (cantons) utilisation-based hospital service areas (HSA) for geographical analyses. HSA are prespecified by the SFSO and reflect the catchment area of a hospital [24]. HSAs and cantons are compatible with small area geographical units used in Switzerland to provide anonymized data on patients’ residences based on small geographical areas. Census data and data on patients’ residences were provided by the SFSO. Each small area is home to approximately 10,000 inhabitants and is contained within a single HSA or canton [25]. Based on discharge data from the Swiss acute care hospitals, HSAs are defined and maintained by the SFSO [24]. Switzerland currently contains 705 small areas, 61 HSAs and 26 cantons.

Using HSAs and small areas is an established approach to analysing area-specific medical data [2]. It ensures compatibility of the medical statistical data for hospitals with census data across all three levels (small areas, HSA, canton).

### Variables and measurements

All variables used are described in the SFSO’s variable specifications for medical statistical data for hospitals and are applicable to the 2017 dataset [26]. Variables include data on diagnosis, locational and sociodemographic characteristics, as well as determinants of hospital stay, admission and discharge (see supplementary file C).

### Statistical procedures

All analyses were performed using R 3.5.2 [27]. The SFSO provided the dataset without missing data of the relevant variables. We performed descriptive analyses on the dataset after filtering out hospitalisations that were referrals from other hospitals or rehabilitation clinics or were patients under the age of 15. The dataset provided information on whether and when each patient was rehospitalised. With these data we calculated rehospitalisation rates within the given year. We also calculated comorbidity scores for each patient using the Elixhauser comorbidity score and the “comorbidity 0.5.3” software package [28, 29]. Used to provide a condensed score for all defined comorbidities for each patient, the Elixhauser comorbidity score theoretically ranges from − 19 to + 89, with higher scores indicating more comorbidities [28, 29].

To determine the number of hospitalisations for each stratum, data were aggregated to each level (small area, HSA, canton), each diagnosis group and care structure (nursing home, home care or home) Rates were calculated using the number of admissions for potentially avoidable hospitalisations as numerator and the population of each small area over the age of 15 as denominator and multiplied by 100,000. The rates for each small area were standardized for sex and age using direct standardisation based on the 2013 standard population for the European Union (EU) [30]. We then calculated median rates of potentially avoidable hospitalisation per 100,000 adult inhabitants, as well as interquartile ranges (IQR) for all ACSCs and care structures for all HSAs (*n* = 61) and cantons (*n* = 26). Outliers - datapoints 1.5 times the IQR above the upper or below the lower quartile -, were assessed individually and kept in the dataset.

For inferential statistical analysis we used linear mixed models with Gaussian distribution to assess regional variation using the “lme4” software package [31]. Intraclass correlation coefficient (ICC 1) were calculated for the HSA and canton levels using the package “RptR” with bootstrap set at 2000 [32]. ICC 1 values above 0.05 were considered meaningful [33]. Models were calculated with random effects for HSAs and cantons.

For geographic visualisation we used SFSO-provided geodata. The “sf 0.8.1” and “tidyverse 1.3.0” software packages were used to merge the geodata with the dataset and compute spatial visualisations [34, 35].

## Results

### Characteristics of potentially avoidable hospitalisations

The data from 2017 included 287 hospitals and specialized clinics that reported to the SFSO. This included all Swiss hospitals [20]. In 2017, SFSO medical statistical data recorded 1,468,245 hospitalisations. Excluding paediatric hospitalisations (< 15 years) and those resulting from referrals from other hospitals or rehabilitation clinics left 1,076,716. From this number, we identified and included 46,479 with main diagnoses corresponding with one of our selected ACSCs, possibly indicating potentially avoidable hospitalisations.

**Fig. 1:**
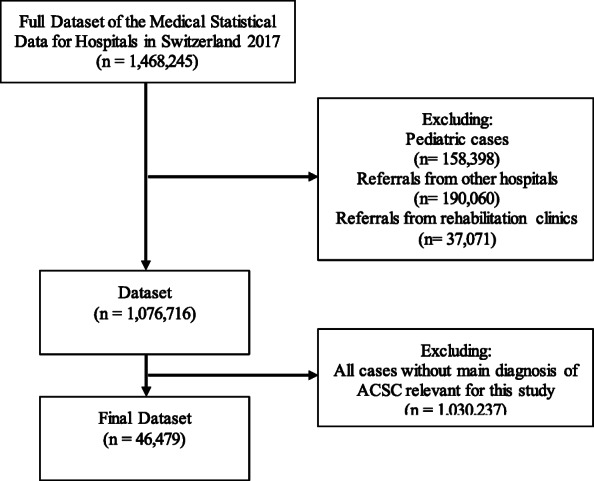
Flow Diagram of Sample Selection Process

**Fig. 2:**
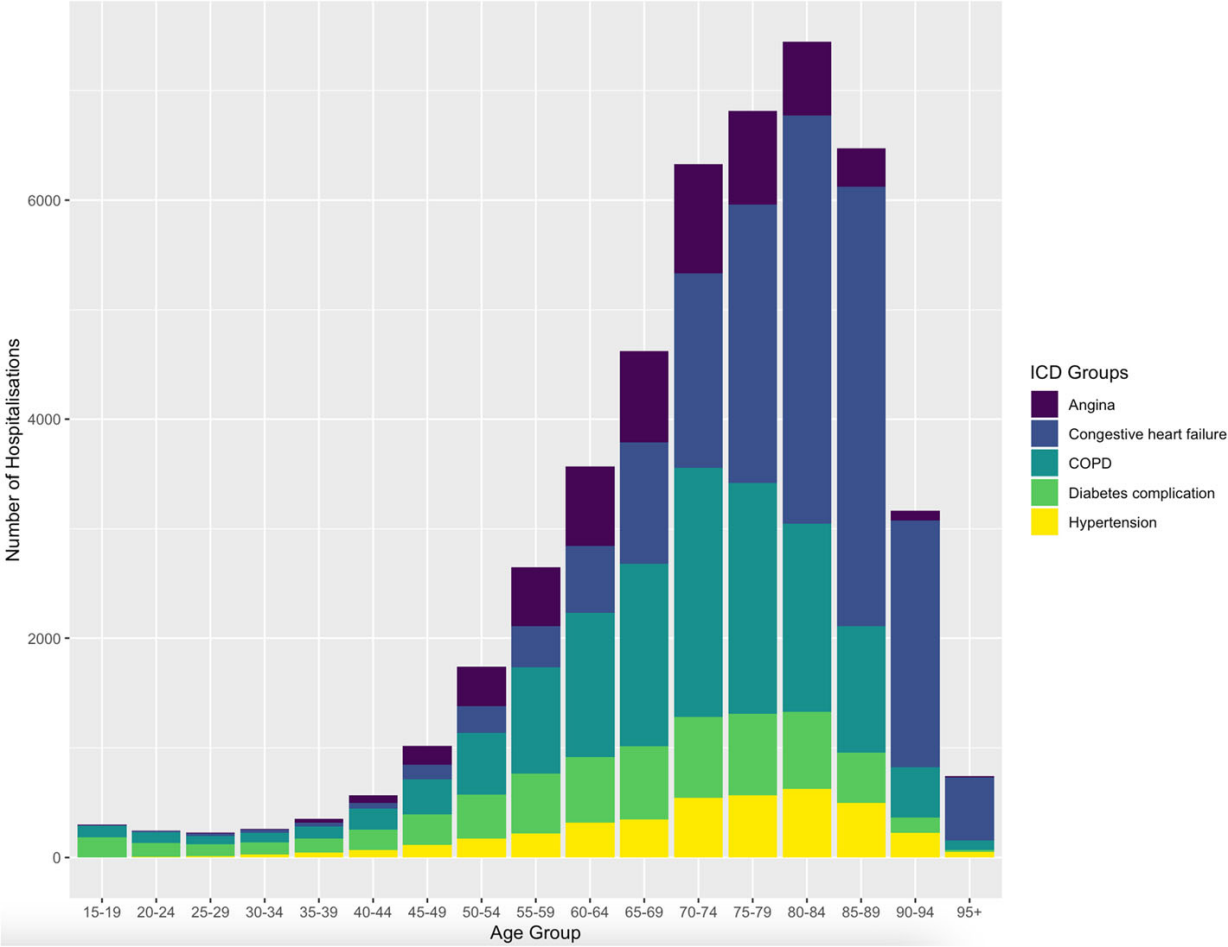
Age Distribution of Hospitalisations and Corresponding Diagnosis

Figure 1 illustrates the sample selection process. Our sample amounted to 4.3% of all hospitalisations from primary health care in the adult population. We observed a median length of stay of 6 (IQR 2–10) days for potentially avoidable hospitalisations for ACSC and a median Elixhauser comorbidity score of 4.5 (IQR 4.0–5.0). Of all hospital admissions for ACSC in 2017, physicians were referring 46.8% of cases and about 28% of cases were self-referrals or by a next of kin. Overall, 78.2% of admissions for were referred as emergencies and 21.5% were scheduled. Mortality rate in ACSC cases was 4.1% and the rehospitalisation rate was 30.0%. The age distribution regarding hospitalisations for ACSCs is illustrated in Fig. 2. We found that 90.2% of such hospitalisations for ACSCs came from home, while 2.1% were patients using home care services. Cases from nursing homes amounted to 4.7% of potentially avoidable hospitalisations for ACSCs and 3% of cases came from psychiatric, penal, other or unknown institutions. For more details on sample characteristics see Tables 2 and 3.

**Table 2:** Sample Characteristics by Diagnosis

	N (%)	Physician Contact prior to Admission n (%)	Rate of Self Referral	Admission as Emergency n (%) *	Scheduled Admission n (%) *	Rehospitalisations (within 2017) n (%)	Mortality n (%)	Median Length of Stay in Days (IQR)	Median Elixhauser Index Score (IQR)
**Cases of PAH for ACSC in Switzerland**	46,479 (100%^a^)	21,763 (46.8% ^a^)	13,040 (28,1% ^a^)	36,353 (78.2% ^a^)	10,016 (21.5% ^a^)	13,952 (30.0% ^a^)	1912 (4.1% ^a^)	6 (2–10)	4.5 (4.0–5.0)
**Angina Pectoris**	5707 (12.3%^a^)	4135 (72.4% ^b^)	947 (16.6% ^b^)	2436 (42.7% ^b^)	3267 (57.2% ^b^)	1486 (26.0% ^b^)	40 (0.7% ^b^)	2 (1–3)	4.0 (4.0–5.0)
**Congestive Heart Failure**	17,514 (37.7%^a^)	7506 (42.9% ^b^)	4880 (27.9% ^b^)	15,472 (88.3% ^b^)	1975 (11.3% ^b^)	5506 (31.4% ^b^)	1379 (7.9% ^b^)	8 (5–13)	5.0 (4.0–5.0)
**COPD**	13,313 (28.6%^a^)	5525 (41.5% ^b^)	4066 (30.5% ^b^)	10,815 (81.2% ^b^)	2473 (18.6% ^b^)	4253 (31.9% ^b^)	407 (3.1% ^b^)	6 (3–9)	4.5 (4.0–5.0)
**Diabetes Complications**	6140 (13.2%^a^)	3354 (54.6% ^b^)	1513 (24.6% ^b^)	4332 (70.5% ^b^)	1796 (29.2% ^b^)	1900 (30.9% ^b^)	80 (1.3% ^b^)	7 (3–11)	4,5 (4.0–5.0)
**Hypertension**	3805 (8.2%^a^)	1243 (32.7% ^b^)	1634 (42.9% ^b^)	3298 (86.7% ^b^)	505 (13.3% ^b^)	807 (21.2% ^b^)	6 (0.2% ^b^)	2 (1–5)	5.0 (4.0–5,0)

Legend: *ACSC* Ambulatory Care Sensitive Condition / *PAH* Potentially Avoidable Hospitalisation / *IQR* Interquartile Range.

^a^ = as percentage of total ACSC Admissions for 2017

^b^ = as percentage of respective subgroup of ACSC admissions for 2017

*Some admissions were coded as “other” and “unknown” thus percentages do not add up to 100%

**Table 3:** Sample Characteristics by Care Structure

	N (%)	Physician Contact prior to Admission n (%)	Admission as Emergency n (%)	Scheduled Admission n (%)	Rehospitalisations (within the Year) n (%)	Mortality n (%)	Median Length of Stay in Days (IQR)	Median Elixhauser Index Scores (IQR)
**Overall cases of PAH for ACSC in Switzerland**	46,479 (100%^a^)	21,763 (46.8% ^a^)	36,353 (78.2% ^a^)	10,016 (21.5% ^a^)	13,952 (30.0% ^a^)	1912 (4.1% ^a^)	6 (2–10)	4.5 (4.0–5.0)
Cases of PAH for ACSC from Home*	41,924 (90.2% ^a^)	19,783 (47.2% ^b^)	32,368 (77.2% ^b^)	9526 (22.7% ^b^)	12,716 (30.3% ^b^)	1546 (3.7% ^b^)	6 (2–10)	4.0 (4.0–5.0)
Cases of PAH for ACSC from Nursing Homes*	2186 (4.7%^a^)	985 (45.1% ^b^)	1925 (88.1% ^b^)	242 (11.1% ^b^)	564 (25.8% ^b^)	228 (10.4% ^b^)	7 (5–12)	4.5 (4.0–5.0)
Cases of PAH for ACSC from Home Care*	972 (2.1%^a^)	365 (37.6% ^b^)	894 (92.0% ^b^)	77 (8.0% ^b^)	367 (37.8% ^b^)	61 (6.3% ^b^)	9 (6–14)	5.0 (4.0–5.0)

Legend: *ACSC* Ambulatory Care Sensitive Condition / *PAH* Potentially Avoidable Hospitalisation / *IQR* Interquartile Range.

^a^ = as percentage of total ACSC Admissions for 2017

^b^ = as a percentage of the respective subgroup of ACSC Admissions for 2017

*Hospitalisations from psychiatric institutions, penitentiaries or other were not included, therefore the numbers of cases do not total 46,479

### Regional variation

For this study, Switzerland was divided into 705 small areas with a median population size of 10,665 (IQR: 8261–14,356). These occupied 61 hospital service areas (HSA) with a median population of 95,353 (IQR: 64,748–163,939). The 26 Swiss cantons provided the highest level for this analysis, with a median population of 234,857 (IQR: 75,384–393,331).

Overall, the median unadjusted rate of potentially avoidable hospitalisation was 489 (IQR 396–592, min. 102, max. 1677) per 100,000 adult inhabitants. The overall sex- and age-standardized median rate of potentially avoidable hospitalisation for ACSC was 527 (IQR 432–620, min. 111, max. 1477) per 100.000 adult inhabitants. On the HSA level the ICC 1 was 0.16 (95% CI: 0.09–0.25); on the cantonal level, it was 0.20 (95% CI: 0.09–0.31).

**Fig. 3:**
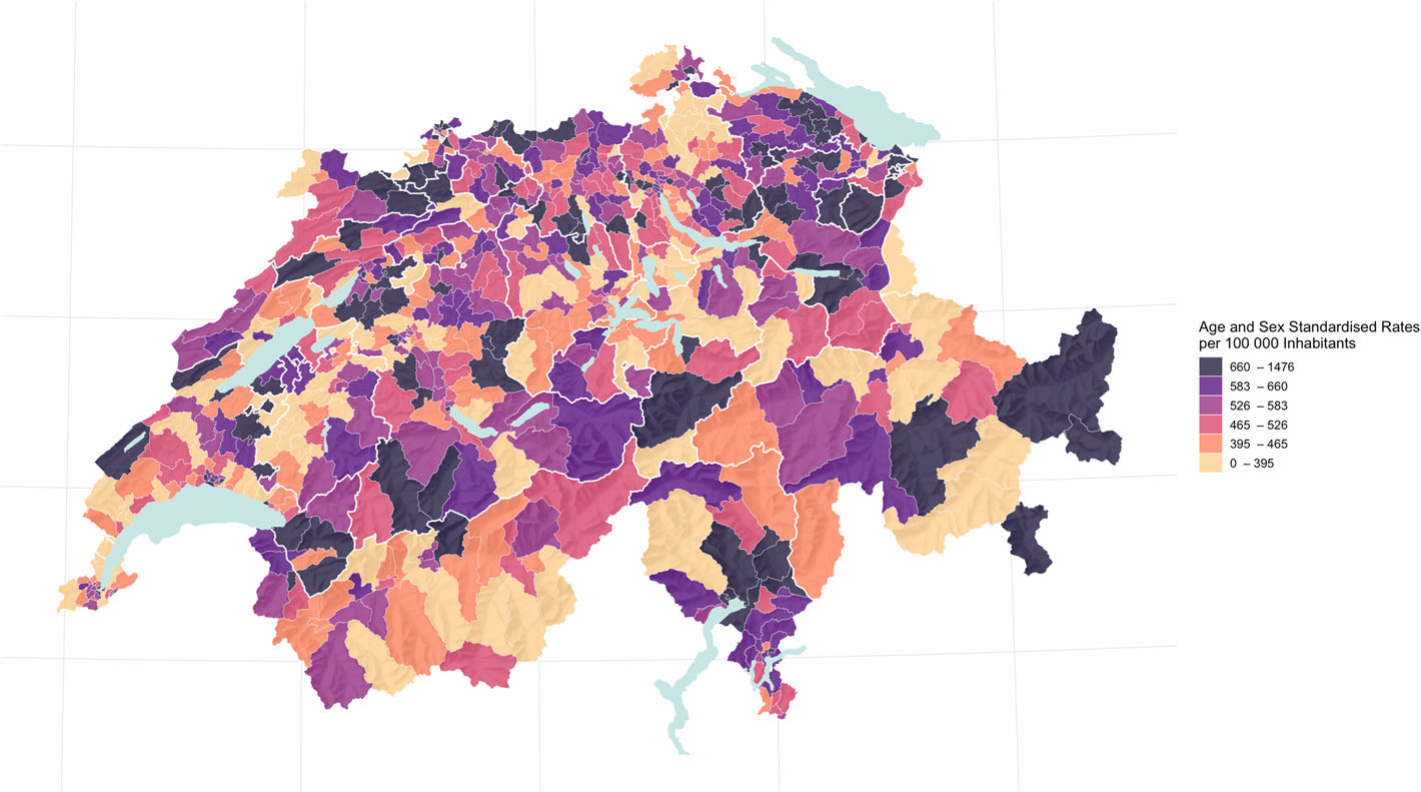
Hospitalisations for all Ambulatory Care Sensitive Conditions in Switzerland in 2017. *Cantonal borders are indicated by bold white lines; lakes appear in blue.*

Table 4 describes unadjusted and direct age- and sex-standardized rates per 100.000 inhabitants for the observed ACSC and respective ICC 1 values for the HSA and cantonal levels. Table 5 describes unadjusted and direct age- and sex-standardized rates per 100,000 inhabitants for the observed settings and respective ICC 1 values for the HSA and cantonal levels. A geographical representation of total sex- and age-standardized rates of potentially avoidable hospitalisation for ACSC per 100,000 adult inhabitants of all 705 small areas is provided in Fig. 3. Additional choropleth maps for the three settings (home, nursing home and home care) and for the various diagnostic groups (angina pectoris, congestive heart failure, COPD, diabetes complications and hypertension) are available in the supplementary materials (Figs. S1, S2, S3, S4, S5, S6, S7, S8).

**Table 4:** Unadjusted and Standardized Rates of Potentially Avoidable Hospitalisation for ACSCs by Diagnosis and Intraclass Correlation Coefficients for the Levels of Analysis

	Unadjusted Median Overall Rate per 100,000 Adult Inhabitants (IQR)	ICC 1 for HSA with Unadjusted Rates (95% CI)	ICC 1 for Canton with Unadjusted Rates (95% CI)	Age and Sex Standardized Median Overall Rate per 100,000 Adult Inhabitants (IQR)	ICC 1 for HSA with Age and Sex Standardized Rates (95% CI)	ICC 1 for Canton with Age and Sex Standardized Rates (95% CI)
**Overall rates of PAH for ACSCs in Switzerland**	**489 (396–592)**	0.29 (0.19–0.39)	0.35 (0.19–0.48)	**527 (432–620)**	0.16 (0.09–0.25)	0.20 (0.09–0.31)
**Angina Pectoris**	**61 (39–90)**	0.47 (0.36–0.57)	0.70 (0.55–0.80)	**59 (38–90)**	0.50 (0.40–0.60)	0.70 (0.54–0.80)
**Congestive Heart Failure**	**166 (125–212)**	0.16 (0.08–0.23)	0.12 (0.05–0.22)	**193 (149–237)**	0.15 (0.07–0.23)	0.11 (0.04–0.21)
**COPD**	**135 (101–185)**	0.24 (0.15–0.33)	0.30 (0.15–0.43)	**145 (107–192)**	0.14 (0.07–0.21)	0.17 (0.07–0.28)
**Diabetes Complications**	**68 (46–92)**	0.17 (0.09–0.25)	0.11 (0.04–0.20)	**67 (46–91)**	0.12 (0.05–0.20)	0.08 (0.02–0.16)
**Hypertension**	**42 (25–62)**	0.32 (0.22–0.42)	0.34 (0.19–0.48)	**39 (21–59)**	0.32 (0.22–0.42)	0.35 (0.19–0.48)

Legend: *95% CI* 95% Confidence Interval / *ACSC* Ambulatory Care Sensitive Condition / PAH = Potentially Avoidable Hospitalisation / *HSA* Hospital Service Area *ICC 1* Intraclass Correlation Coefficient 1 / *IQR* Interquartile Range.

**Table 5:** Unadjusted and Standardized Rates of Potentially Avoidable Hospitalisations for ACSC by Care Structure and Intraclass Correlation Coefficients for the Levels of Analysis

	Unadjusted Median Overall Rate per 100,000 Adult Inhabitants (IQR)	ICC 1 for HSA with Unadjusted Rates (95% CI)	ICC 1 for Canton with Unadjusted Rates (95% CI)	Age and Sex Standardized Median Overall Rate per 100,000 Adult Inhabitants (IQR)	ICC 1 for HSA with Age and Sex Standardized Rates (95% CI)	ICC 1 for Canton with Age and Sex Standardized Rates (95% CI)
**Overall rates of PAH for ACSC in Switzerland**	**489 (396–592)**	0.29 (0.19–0.39)	0.35 (0.19–0.48)	**527 (432–620)**	0.16 (0.09–0.25)	0.20 (0.09–0.31)
**Rates of PAH for ACSC from Home**	**468 (385–571)**	0.32 (0.21–0.41)	0.38 (0.22–0.51)	**475 (393–564)**	0.19 (0.11–0.28)	0.26 (0.13–0.39)
**Rates of PAH for ACSC from Nursing Homes**	**19 (5–39)**	0.18 (0.10–0.27)	0.17 (0.07–0.27)	**18 (0–36)**	0.16 (0.09–0.25)	0.13 (0.05–0.24)
**Rates of PAH for ACSC from Home Care**	**0 (0–16)**	0.51 (0.39–0.60)	0.28 (0.15–0.41)	**0 (0–15)**	0.52 (0.40–0.61)	0.34 (0.19–0.49)

Legend: *95% CI* 95% Confidence Interval / *ACSC* Ambulatory Care Sensitive Condition / *PAH* Potentially Avoidable Hospitalisation / *HSA* Hospital Service Area *ICC 1* Intraclass Correlation Coefficient 1 / *IQR* Interquartile Range.

## Discussion

This study provides the first complete mapping of potentially avoidable hospitalisations for ACSCs in Switzerland in 2017. Using small area analysis to determine regional variation for various ACSCs and primary care structures, we found substantial regional variation with distinct disease-specific regional patterns. Standardized for sex and age, the overall degree of regional variation was higher than in other European countries i.e. Denmark, England Portugal, Slovenia and Spain [36].

### General characteristics

Our results suggest that up to 4.3% of all hospitalisations in 2017 were avoidable. Furthermore, we observed a gradual increase in potentially avoidable hospitalisations for ACSCs in the population above 65 years of age, peaking at the 80–84-year age group. This pattern is consistent with results from a similar study that investigated potentially avoidable hospitalisations for ACSCs in France [6].

More specifically, consistent with previous studies in Germany, we found that hospitalisations for congestive heart failure and COPD account for a substantial fraction of potentially avoidable hospitalisations [7, 10]. About half of the identified cases were referred to hospital by physicians, with roughly three quarters of patients admitted to hospital as emergencies. Interestingly 21,5% of admissions for ACSC were scheduled. These were still considered potentially avoidable as it remains unclear as to how many days in advance this admission was scheduled and whether they could have been prevented with adequate ambulatory care.

Our sample’s Elixhauser comorbidity scores were rather high at 4.5 (IQR 4.0–5.0) compared to those measured by van Walraven et al. (2009), who recorded a median score of 0 (IQR 0–8). This indicates, that our population had more comorbidities present, than a regular hospital cohort. Additionally, the high number of emergency admissions suggest that the admitted patients had experienced a profound deterioration of their already fragile health prior to admission with multiple comorbidities. Comorbidities within different age groups and admission types could be analysed in further research to better understand contributing factors of variation. Interestingly, about 30% of cases are readmitted to hospital within the year 2017, indicating challenges in primary health care provision, especially regarding self-management and monitoring of early warning signs.

When addressing overall unadjusted rates of potentially avoidable hospitalisations for ACSCs, we found similar results for four of our diagnoses of interest (congestive heart failure, COPD, diabetes complications and hypertension) also used in a similar study by Berlin et al. (2014) in the Swiss context [2]. Compared with that study’s findings, our overall unadjusted rates of potentially avoidable hospitalisations for ACSCs indicate an increase of 2.7% over a 7 year period [2]. However, compared to similar studies in Swiss, French and German contexts [2, 6, 7], this increase is actually quite low.

Standardized rates of potentially avoidable hospitalisations for ACSCs in Switzerland were comparable to other European countries; likewise, regional variation for the ACSCs of interest was considerably higher in Switzerland than in other European countries [36]. The difference between raw and standardized rates of potentially avoidable hospitalisations for ACSCs in Switzerland is due to a relatively high number of cases in the age group 60–79. That means that due to relatively smaller Swiss population in these age groups compared to the EU standard population, these cases lead to higher standardized rates.

### Regional variation

The findings suggest a high degree of variation amongst HSA and cantons regarding potentially avoidable hospitalisations for all ACSCs. ICC 1 values for all settings and diagnosis groups where meaningful both for unadjusted and standardized rates with variation being substantially lower with standardized rates. Therefore, considerable variation was reduced due to differences in demographics.

We found pronounced geographical patterns based on both diagnosis and setting. Most prominently, variation in the management of angina pectoris shows substantial variation in Switzerland’s northern and north-eastern regions. Interestingly, when assessing rates of congestive heart failure, these patterns shift towards the southwest. Regarding hypertension, though, we found consistently lower rates in the southwest. Moreover, we found a substantial amount of variation amongst the diagnosis groups in regard to the mode of referral. Especially for self-referrals it would be of interest to determine, whether these differences arise from hospital supply inducement or factors associated with ambulatory care provision. While the patterns found indicate some of the challenges HSAs and cantons face in providing specialized primary health care for different diseases, they also underscore the importance of differentiating between diseases and visualizing results to address issues in primary health care provision.

The broad regional variation for the various diagnoses may reflect specific regional (cantonal) and municipal approaches to primary care provision. Intra-cantonal variation was considerable mostly in regions with a strong urban rural gap. Evidence supports the possibility that socioeconomic, demographic and provider specific determinants, such as hospital supply inducement, contribute to the emergence of potentially avoidable hospitalisations for ACSCs [13, 17, 37–41]. While it remains unclear just how these factors affect the rates of ACSC-related hospitalisation, minimizing those rates will demand an understanding of the contributing factors.

### Contributing factors and impulse for health policy

Evidence suggests that physician density, healthcare accessibility, resources for primary health care and continuity of care are all related to rates of potentially avoidable hospitalisation for ACSCs [2, 42–46]. In Switzerland, except in some isolated alpine regions, accessibility to primary health care is consistently high [47], i.e., resources for primary health care were reinforced in 2014, and physician density is sufficient. More physicians might actually lower healthcare efficiency: several studies suggest that high physician density can inflate demand for health care services [2, 40, 48]. However, one must note that it is not only physician density but utilisation is relevant. A recent study found that rates of hospitalisations for ACSC steadily decreases with additional medical services present, with diminishing marginal returns. This raises the question as to how high rates of potentially avoidable hospitalisation for ACSCs despite high accessibility to and strong resourcing for primary care can be explained. These additional medical services might only have an effect some regions. Moreover, a recent study in Germany the most prominent reason for variation in hospital utilisation were medical needs [49]. More services must therefore be aligned with the medical needs of the population. Our analysis focussed on chronic condition where continuity of care is crucial. Continuity of care should focus on a team-based approaches to reduce fragmentation of care and improve patient safety and quality of care [50]. Chronic care management in Switzerland is still predominately provided by primary care physicians. With Switzerland’s primary care physician workforce aging this may eventually lead to a shortage of general practitioners and disrupt chronic care management [16]. Additional necessary medical services in chronic care management for specific populations might therefore require novel roles in care delivery for the chronically ill [51]. Interventions to reduce hospitalisations for ACSC include specialized home care, promotion of self-management and the integration of primary and secondary care [52]. Swiss health policy makers could address these challenges by bridging the gap in chronic care management.

The geographical representation and small area approach differentiated by diagnosis and care structure highlight the various Swiss regions’ relative success at minimizing potentially avoidable hospitalisations. There is a need to understand the specific context and its impact on potentially avoidable hospitalisations. Health policy makers should address these regional variations with a distinct focus on strengthening care management for the chronically ill.

### Strengths and limitations

This study offers the first complete map of potentially avoidable hospitalisations for ACSCs in Switzerland. There are, however, several limitations. Selection criteria for ACSCs differ in the literature and interpretations differ regarding the preventability of certain ACSC-related hospitalizations [5]. Moreover, we cannot discriminate between clinically avoidable or necessary hospitalisations beyond the information provided within the routine dataset. Further, this study did not account for sociodemographic or socioeconomic differences such as education and income. Nor did it examine behavioural or cultural factors affecting the use of hospitals and primary care or account for the distribution of healthcare structures, e.g. the number of nursing homes within an area. Units in this study where provided by the SFSO using prespecified geographical areas, thus limiting impact of the modifiable areal unit problem on our analysis. However, we cannot rule out this bias. Still, the study offers a new perspective on regional variation of potentially avoidable hospitalisations for ACSCs in Switzerland. On the other hand, one of this study’s strengths is the inclusion of the most prevalent ACSCs for chronic conditions. This will help first to identify well-functioning primary care services in regions to inform and enable health policy adjustments.

## Conclusion

This study identified substantial regional variation in and comparably high rates of avoidable ACSC-based hospitalisation in Switzerland. We suspect that differences in continuity of care are predominantly responsible for these regional variations. As ACSCs account for an increasing number of hospitalisations in Switzerland, indicating a need for multidisciplinary care models of care that allow increased continuity of care, they should be dealt with specifically at the health policy level. Further research is needed to model and assess the impact of different primary care models on ACSCs.

## Supplementary Information

**Additional file 1:** Additional definitions of terms in the Swiss Setting.

**Additional file 2: Supplementary file B**-List of abbreviations.

**Additional file 3: Supplementary file C. Table S1**. Summary of variables and measurements.

**Additional file 4: Fig. S1**. Hospitalisations for Angina Pectoris in Switzerland in 2017.

**Additional file 5: Fig. S2**. Hospitalisations for Congestive Heart Failure in Switzerland in 2017.

**Additional file 6: Fig. S3**. Hospitalisations for COPD in Switzerland in 2017.

**Additional file 7: Fig. S4**. Hospitalisations for Diabetes Complications in Switzerland in 2017.

**Additional file 8: Fig. S5**. Hospitalisations for Hypertension in Switzerland in 2017.

**Additional file 9: Fig. S6**. Hospitalisations from Home in Switzerland in 2017.

**Additional file 10: Fig. S7**. Hospitalisations from Nursing Homes in Switzerland in 2017.

**Additional file 11: Fig. S8**. Hospitalisations from Home Care in Switzerland in 2017.

## Data Availability

The data that support the findings of this study are available from the Federal Statistical Office of Switzerland. While restrictions apply to the availability of these data, which were used under license for the current study, they are available from the authors upon reasonable request and with permission of the Federal Statistical Office of Switzerland.
