# Supplementary material for: Regional variation of potentially avoidable hospitalisations in Switzerland: an observational study
Source: BMC Health Serv Res. 2021 Aug 21;21:849. doi: 10.1186/s12913-021-06876-5 (PMC8380390; doi:10.1186/s12913-021-06876-5)
Supplement: Supplementary file 1 — Additional file 1: Additional definitions of terms in the Swiss Setting. [file 12913_2021_6876_MOESM1_ESM.docx]

## **A - Additional Definitions of Terms in the Swiss Setting**

Hospitalisations. Hospitalisations are defined by the Swiss Federal Statistical Office (SFSO) as “(…) all treatments, examinations or nursing care provided for at least 24 hours in a hospital bed as inpatient. Furthermore, emergencies that lead to hospitalisation, referrals from another hospital and deaths within a timeframe smaller than 24 hours also qualify as inpatient.”

Long-Term Care Setting. The Swiss long-term care setting consists of 1,561 NHs, with 99,242 beds and 2,035 home care providers with services for 350,218 residents in 2017. NHs are defined by the SFSO as “ institutions that provide long term stays for elderly and or chronically ill people who need care.”. Home care providers are defined as “Outpatient care services provided outside of a hospital or nursing home with nursing care and consultation at the home of a patient.”

Small area. The SFSO utilizes a system called MedStat to geographically segment Switzerland into residential regions large enough to guarantee anonymity of hospitalized patients. These small area clusters of roughly 10,000 residents are aligned with the corresponding postal codes.
