# Supplementary material for: Regional variation of potentially avoidable hospitalisations in Switzerland: an observational study
Source: BMC Health Serv Res. 2021 Aug 21;21:849. doi: 10.1186/s12913-021-06876-5 (PMC8380390; doi:10.1186/s12913-021-06876-5)
Supplement: Supplementary file 2 — Additional file 2: Supplementary file B-List of abbreviations. [file 12913_2021_6876_MOESM2_ESM.docx]

# Supplementary file B

## **List of abbreviations**

ACSC Ambulatory Care Sensitive Conditions

PAH Potentially Avoidable Hospitalisations

COPD Chronic Obstructive Pulmonary Disease

HSA Utilisation-based Hospital Service Areas

ICC 1, 2 Intraclass Correlation 1, 2

ICD-10 International Classification of Disease 10

IQR Interquartile Range

Small area Regional cluster of approximately 10 000 Inhabitants called MedStat

OECD Organisation for Economic Co-operation and Development

SwissDRG Swiss Diagnostic Related Groups (reimbursement system for medical services)

SFSO Swiss Federal Statistical Office

ZIP-Code Zone Improvement Plan Code
