# Supplementary material for: Regional variation of potentially avoidable hospitalisations in Switzerland: an observational study
Source: BMC Health Serv Res. 2021 Aug 21;21:849. doi: 10.1186/s12913-021-06876-5 (PMC8380390; doi:10.1186/s12913-021-06876-5)
Supplement: Supplementary file 3 — Additional file 3: Supplementary file C. Table S1. Summary of variables and measurements. [file 12913_2021_6876_MOESM3_ESM.docx]

**Supplementary file C**

| **Table 1**  *Summary of Variables and Measurements* | | | |  |
| --- | --- | --- | --- | --- |
| **Variable** | **Options/Measurement** | | | **Remarks** |
| *Geographic variables* | |  |  |  |
| Canton | Factor with 26 levels, all Swiss cantons | | | - |
| Hospital service areas (HSA) | Factor with 61 levels | | |  |
| Small area | Factor with 705 levels, place of residence in a subarea of a Swiss canton/HSA | | | ~10 000 inhabitants in each subarea |
| *Sociodemographic variables* |  | | |  |
| Gender | Factor with 2 levels | | |  |
| Age at admission | 5-year age groups (15-19; 20-24; 25-29; …) | | |  |
| Main diagnosis | ICD-10-GM Code | | | Main reason for admission upon discharge |
| Supplementary information for main diagnosis | ICD-10-GM Code | | | Additional information |
| Secondary diagnosis and supplementary information | ICD-10-GM Code | | |  |
| *Characteristics of admission* | |  |  |  |
| Place of residence prior to admission | Factor with 13 levels (home, home with home care, nursing home, residential care, psychiatric facility, psychiatric facility in same institution, other hospital or birth house, ward in same institution, penal institution, other, unknown, rehabilitation clinic, other institution/ same institution) | | | Where was the patient prior to admission? |
| Type of admission | Factor with 7 levels (emergency, scheduled admission, birth, internal referral, referral from other clinic within 24 hours, other, unknown) | | | How was the patient admitted?  Birth means that child is born at institution |
| Referred by … | Factor with 8 levels (self, next of kin, rescue service (police, ambulance), physician, non-medical profession, sociomedical services, justice department, other, unknown) | | | Who initiated the referral to hospital? |
| *Characteristics of stay* | |  |  |  |
| Length of stay (OECD) | In days | | | Length of stay incl. day of admission |
| *Characteristics of discharge* | |  |  |  |
| Decision for discharge | Factor with 7 levels (initiative of care providers, initiative of patient, initiative of third party, internal referral, deceased, other, unknown) | | | Who initiated the discharge? |
| Place of residence after discharge | Factor with 13 levels (home, nursing home, residential care, psychiatric facility, psychiatric facility in same institution, rehabilitation clinic, other institution/ same institution, other hospital or birth house, ward in same institution, penal institution, other, unknown, death) | | | Where was the patient discharged to? |
| Treatment after discharge | Factor with 8 levels (no treatment necessary, ambulatory care, ambulatory nursing care (e.g., home care), inpatient care, rehabilitation (in - /outpatient), other, unknown, death | | |  |
| Days to next hospitalisation | In days | | | Within the year 2017 |
