# Supplementary figures and images for: Regional variation of potentially avoidable hospitalisations in Switzerland: an observational study
Source: BMC Health Serv Res. 2021 Aug 21;21:849. doi: 10.1186/s12913-021-06876-5 (PMC8380390; doi:10.1186/s12913-021-06876-5)

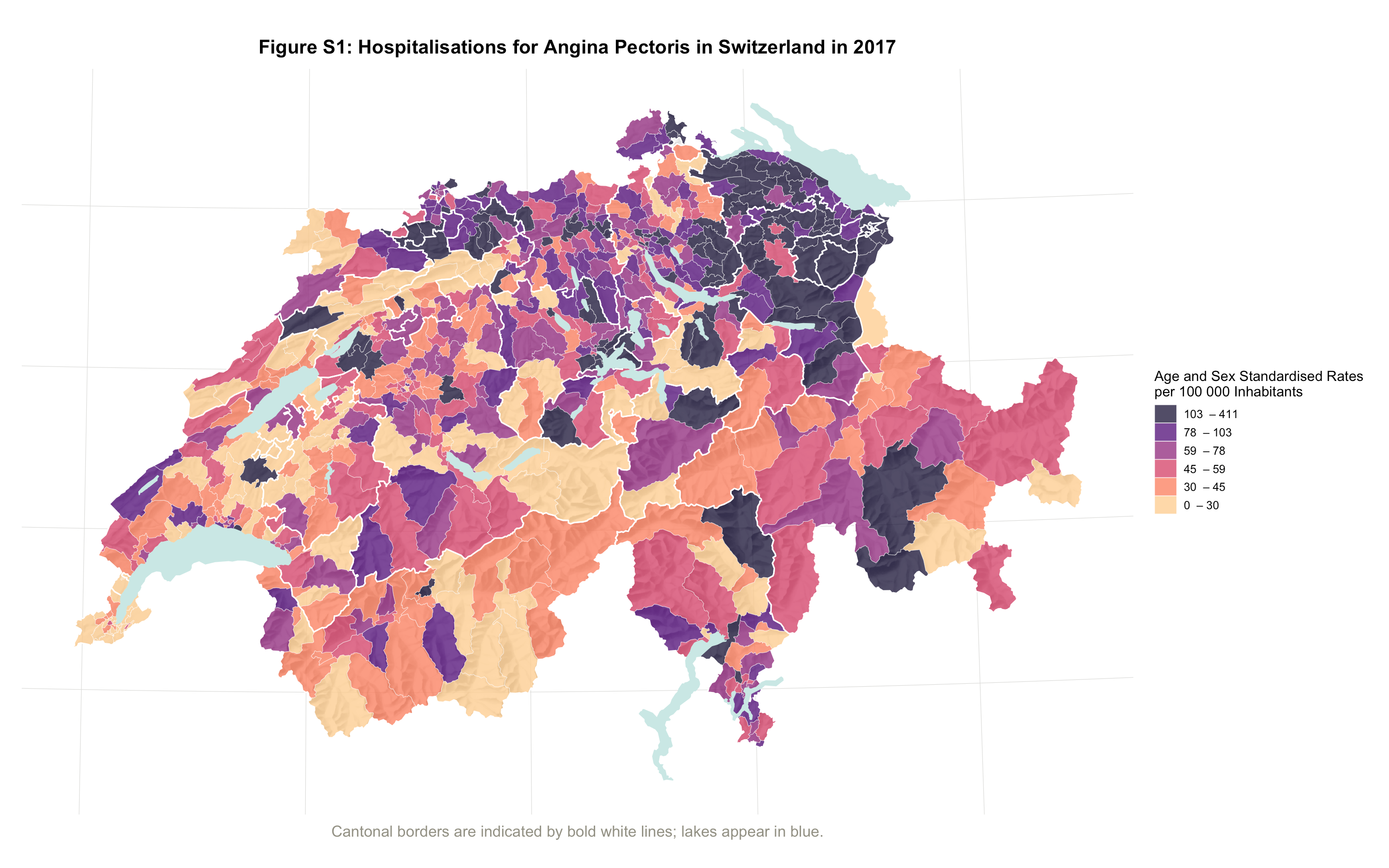

Supplement: Supplementary file 4 — Additional file 4: Fig. S1. Hospitalisations for Angina Pectoris in Switzerland in 2017. [file 12913_2021_6876_MOESM4_ESM.png]

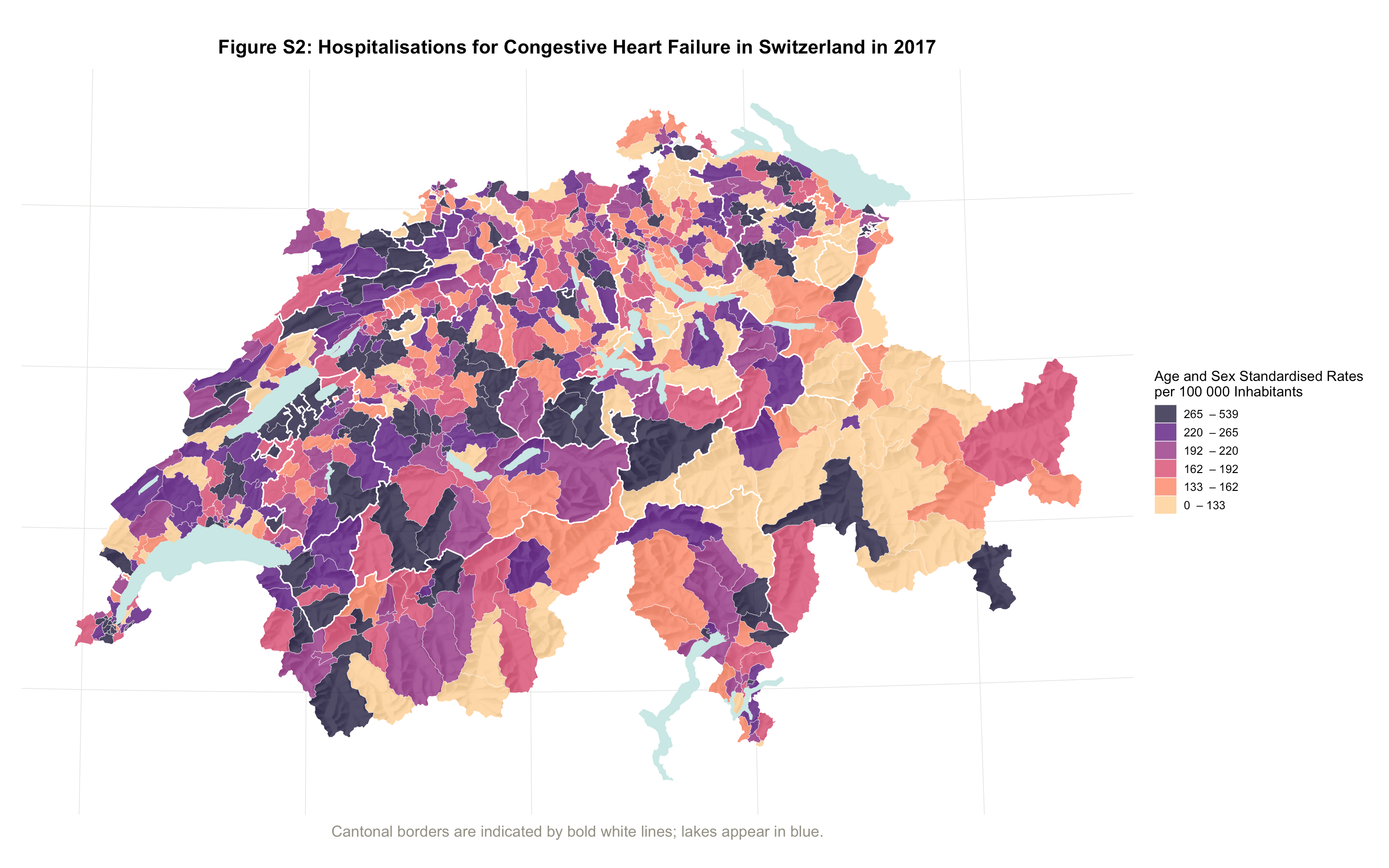

Supplement: Supplementary file 5 — Additional file 5: Fig. S2. Hospitalisations for Congestive Heart Failure in Switzerland in 2017. [file 12913_2021_6876_MOESM5_ESM.png]

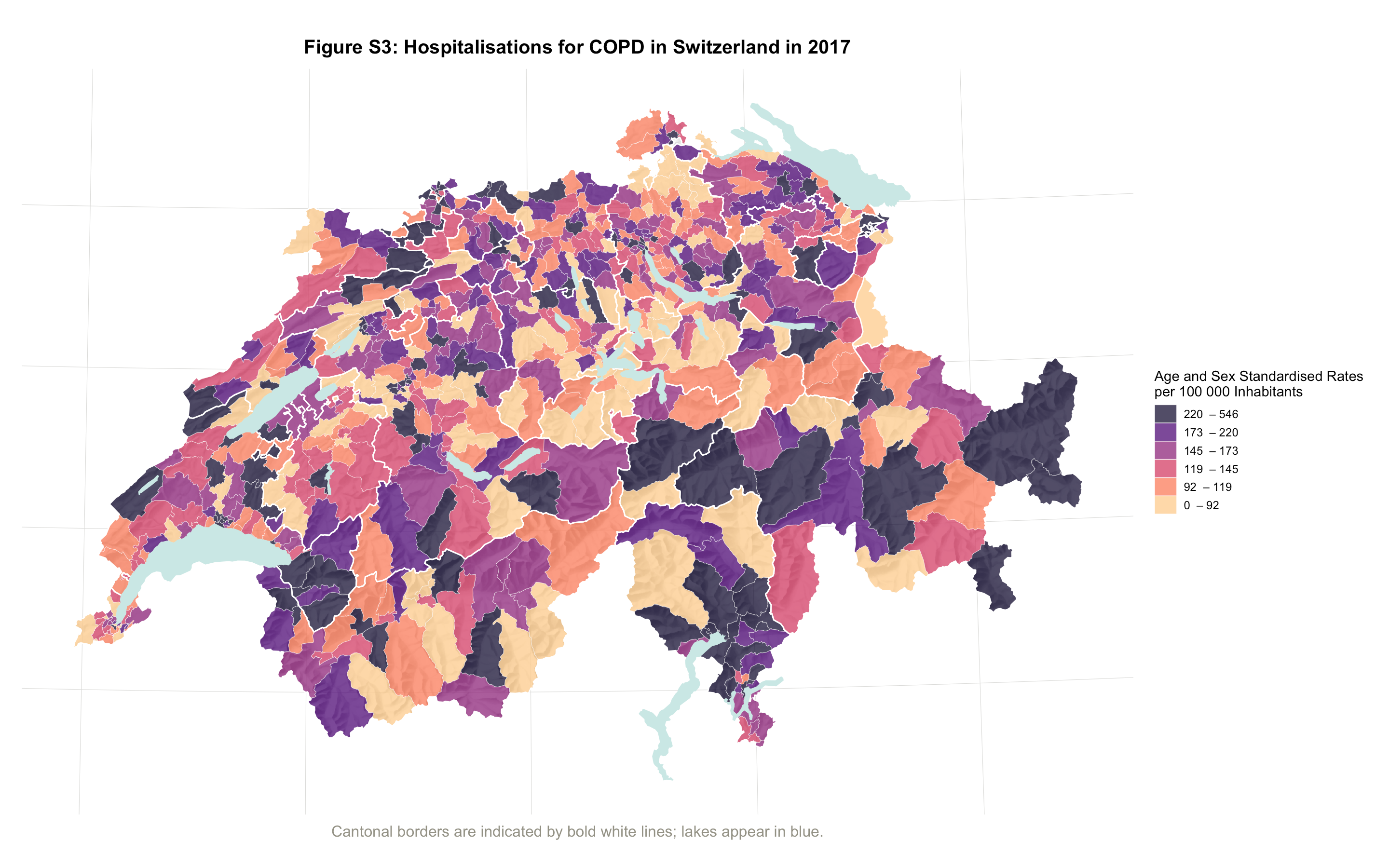

Supplement: Supplementary file 6 — Additional file 6: Fig. S3. Hospitalisations for COPD in Switzerland in 2017. [file 12913_2021_6876_MOESM6_ESM.png]

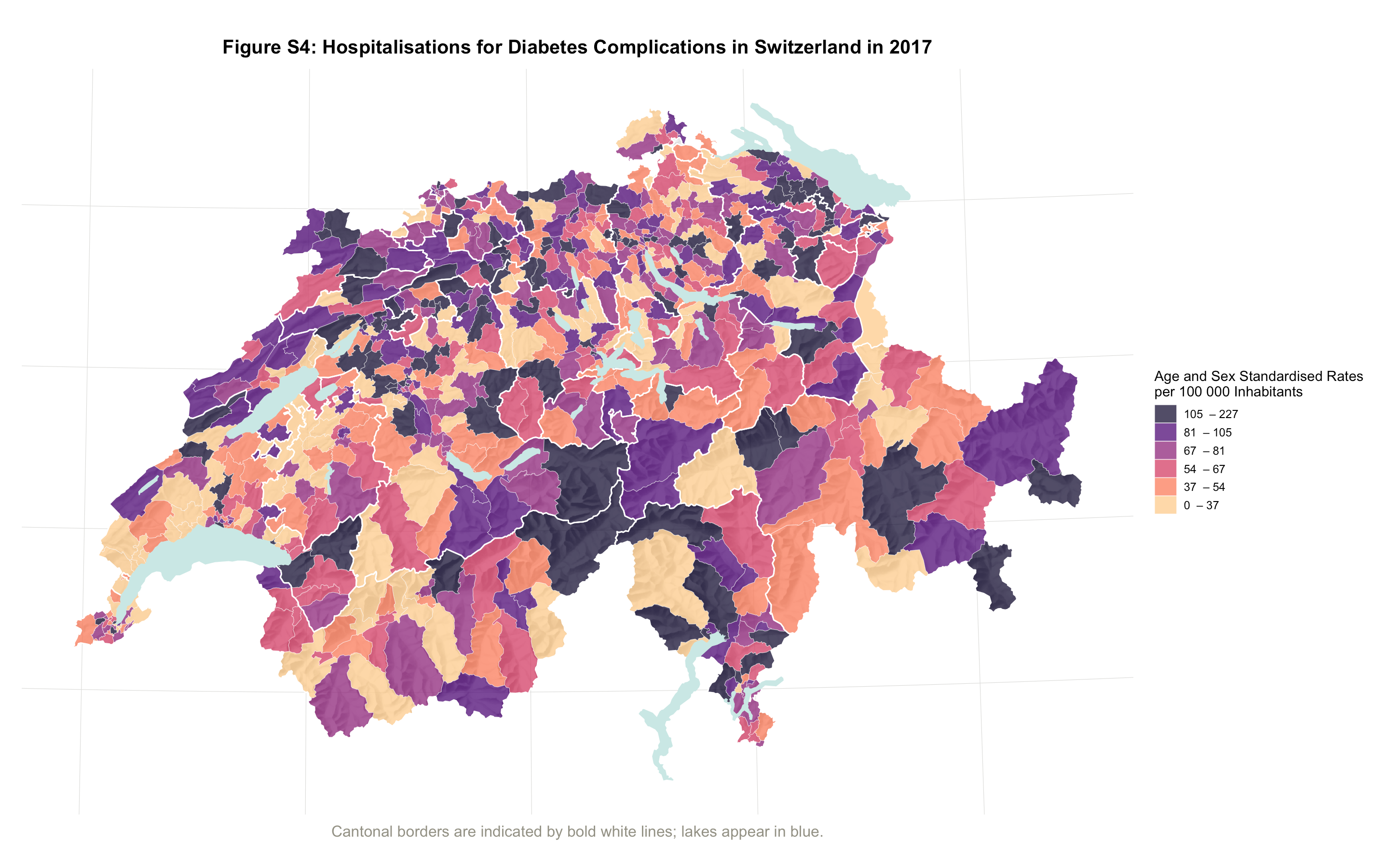

Supplement: Supplementary file 7 — Additional file 7: Fig. S4. Hospitalisations for Diabetes Complications in Switzerland in 2017. [file 12913_2021_6876_MOESM7_ESM.png]

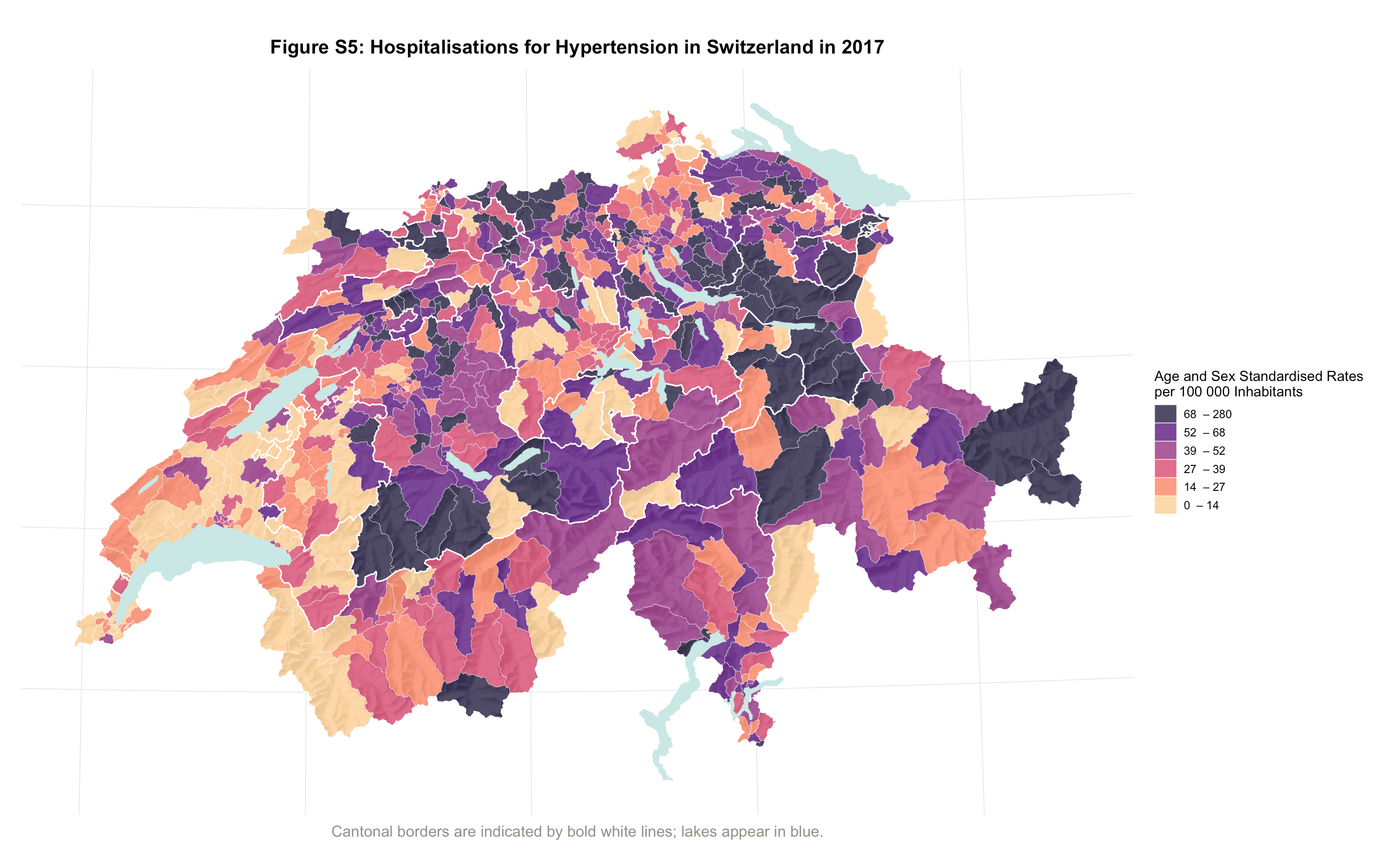

Supplement: Supplementary file 8 — Additional file 8: Fig. S5. Hospitalisations for Hypertension in Switzerland in 2017. [file 12913_2021_6876_MOESM8_ESM.png]

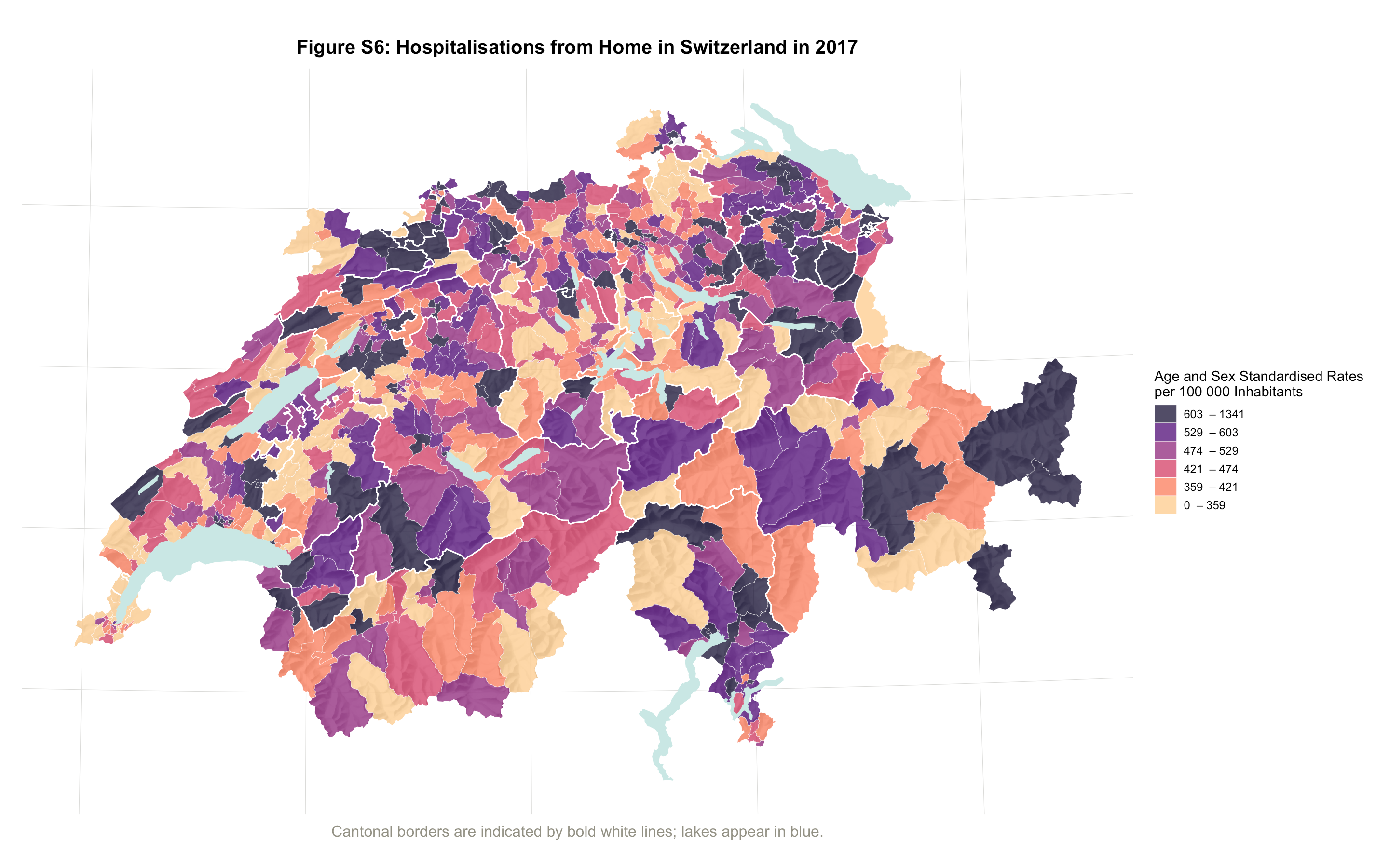

Supplement: Supplementary file 9 — Additional file 9: Fig. S6. Hospitalisations from Home in Switzerland in 2017. [file 12913_2021_6876_MOESM9_ESM.png]

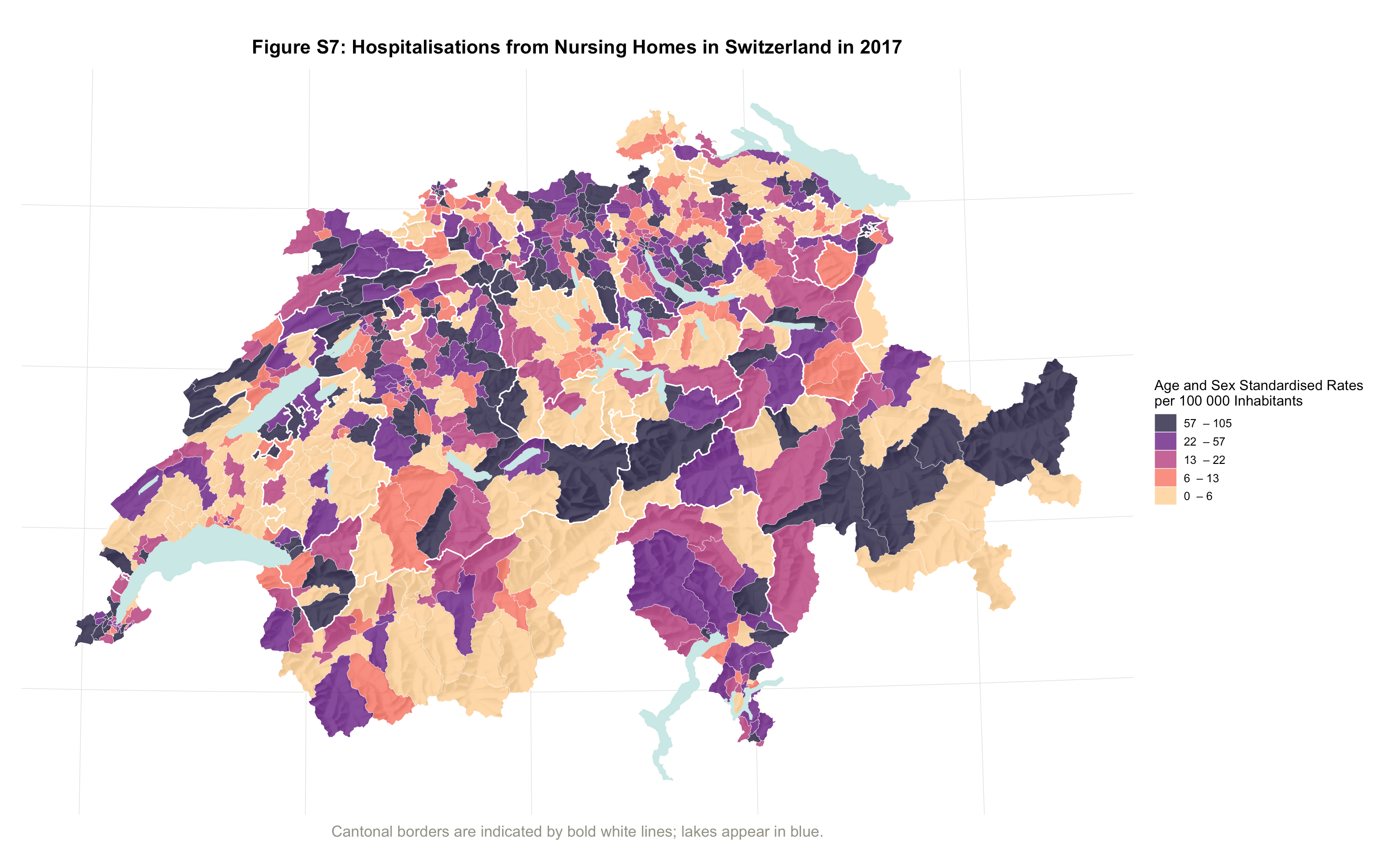

Supplement: Supplementary file 10 — Additional file 10: Fig. S7. Hospitalisations from Nursing Homes in Switzerland in 2017. [file 12913_2021_6876_MOESM10_ESM.png]

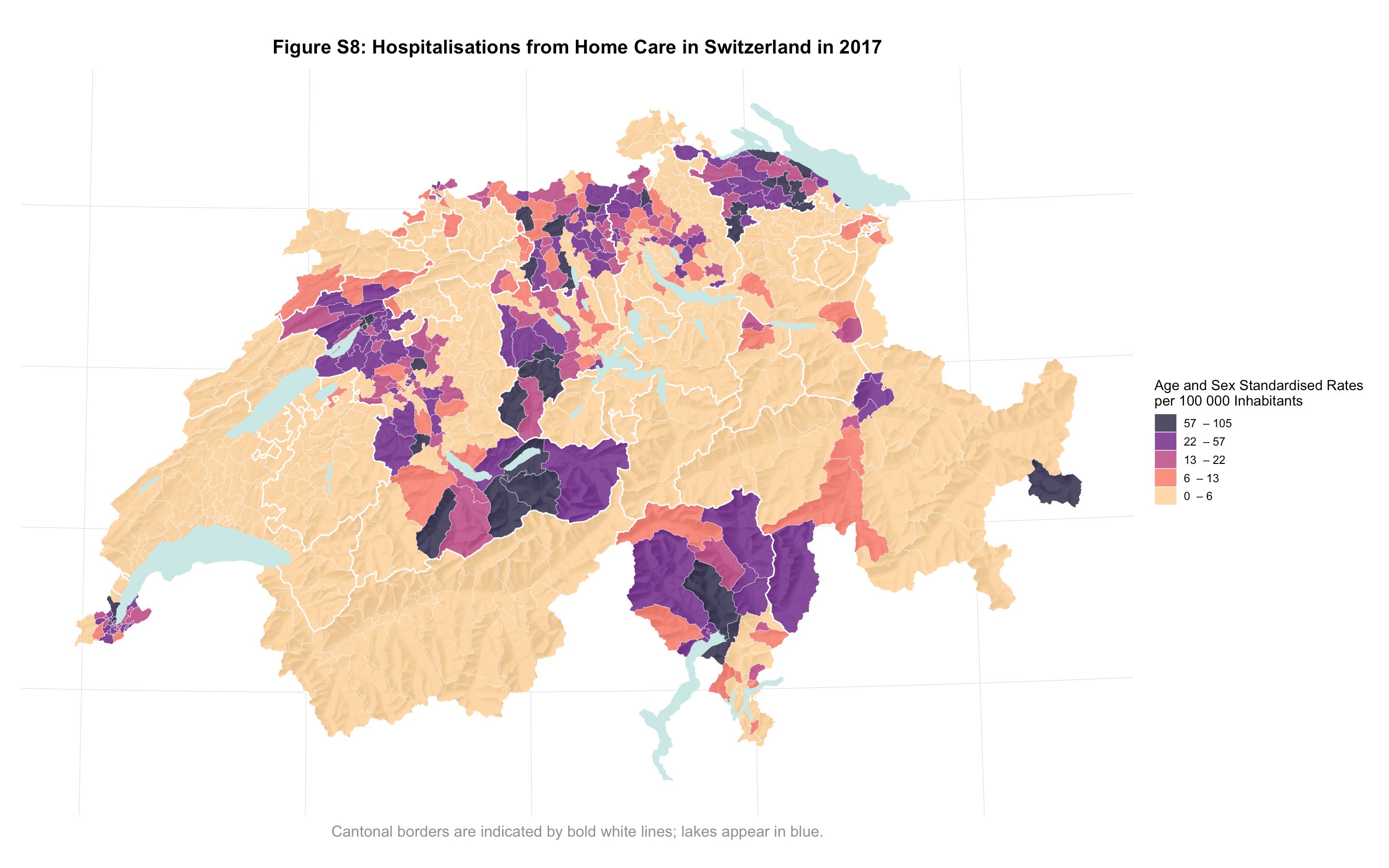

Supplement: Supplementary file 11 — Additional file 11: Fig. S8. Hospitalisations from Home Care in Switzerland in 2017. [file 12913_2021_6876_MOESM11_ESM.png]
